# Characterizing brain structures associated with plasma neurofilament light chain among older adults: variations by sex and APOE gene

**DOI:** 10.1002/alz.088199

**Published:** 2025-01-09

**Authors:** Yi Dong, Lin Song, Tingting Hou, Yuanjing Li, yongxiang wang, Yifeng Du, Chengxuan Qiu

**Affiliations:** ^1^ Shandong Provincial Hospital affiliated to Shandong First Medical University, Jinan China; ^2^ The Swedish School of Sport and Health Sciences, GIH, Stockholm, Sweden., Stockholm Sweden; ^3^ Aging Research Center, Karolinska Institutet and Stockholm University, Stockholm Sweden

## Abstract

**Background:**

Plasma neurofilament light (NfL) has emerged as a promising biomarker to track neurodegeneration in Alzheimer’s disease (AD) and other brain degenerative disorders. We sought to characterize brain structures and white matter lesions associated with plasma NfL among dementia‐free older adults, while taking into account demographic factors and genetic susceptibility (e.g., APOE gene).

**Method:**

This population‐based cross‐sectional study included 844 non‐demented older adults (age ≥60 years, 59.2% women), derived from participants in the baseline assessments of the Multimodal INterventions to delay Dementia and disability in rural China (MIND‐China) study who had plasma NfL measured on the SIMOA platform in 2018 and undertook structural brain magnetic resonance imaging (MRI) scan on a 3T scanner in 2018‐2020. Structural brain measures were assessed using the Computational Anatomy Toolbox on the platform of MATLAB. Data were analysed using the multivariable linear regression models.

**Result:**

Per 1‐SD increase in log‐transformed NfL concentration was significantly associated with multivariable‐adjusted β‐coefficient (95% confidence interval) of 0.09 (0.05 to 0.14) for white matter hyperintensity (WMH) volume, 0.09 (0.05 to 0.14) for periventricular WMH volume, ‐2.71 (‐4.99 to ‐0.43) for grey matter volume, ‐0.03 (‐0.06 to ‐0.01) for entorhinal cortex volume, ‐0.04 (‐0.07 to ‐0.01) for parahippocampal volume, ‐0.22 (‐0.37 to ‐0.07) for AD‐signature volume, and ‐0.02 (‐0.03 to ‐0.01) for AD‐signature cortical thickness. The associations of plasma NfL concentration with the MRI markers of neurodegeneration were statistically evident mainly among males (P for plasma NfL×sex interaction<0.05) or APOE ε4 allele carriers (P for plasma NfL×APOE ε4 allele interaction<0.05).

**Conclusion:**

Brain structures associated with increased plasma NfL in older adults are characterized by reduced grey matter volume, entorhinal cortex volume, parahippocampal volume, and AD‐signature volume, especially among men or carriers of the APOE ε4 allele.

